# Overexpression of Soybean *GmWRI1a* Stably Increases the Seed Oil Content in Soybean

**DOI:** 10.3390/ijms23095084

**Published:** 2022-05-03

**Authors:** Zhikun Wang, Yuanzhuo Wang, Ping Shang, Chao Yang, Mingming Yang, Jinxiu Huang, Baizheng Ren, Zhaohui Zuo, Qingyan Zhang, Wenbin Li, Bo Song

**Affiliations:** Key Laboratory of Soybean Biology of Ministry of Education China, Key Laboratory of Soybean Biology and Breeding (Genetics) of Ministry of Agriculture and Rural Affairs, Northeast Agricultural University, Harbin 150030, China; zhikunwang1998@aliyun.com (Z.W.); wangyuanzhuo404@163.com (Y.W.); 15684178680@163.com (P.S.); yangchao202204@163.com (C.Y.); yangmingming2013@163.com (M.Y.); hjinxiu0320@163.com (J.H.); rbaizheng@163.com (B.R.); zhaohuizuo0519@163.com (Z.Z.); zhangqingyan588@163.com (Q.Z.)

**Keywords:** soybean, *GmWRI1a*, overexpression, oil content, fatty acid composition

## Abstract

WRINKLED1 (WRI1), an APETALA2/ethylene-responsive-element-binding protein (AP2/EREBP) subfamily transcription factor, plays a crucial role in the transcriptional regulation of plant fatty acid biosynthesis. In this study, *GmWRI1a* was overexpressed in the soybean cultivar ‘Dongnong 50’ using *Agrobacterium*-mediated transformation to generate three transgenic lines with high seed oil contents. PCR and Southern blotting analysis showed that the T-DNA was inserted into the genome at precise insertion sites and was stably inherited by the progeny. Expression analysis using qRT-PCR and Western blotting indicated that *GmWRI1a* and *bar* driven by the CaMV *35S* promoter were significantly upregulated in the transgenic plants at different developmental stages. Transcriptome sequencing results showed there were obvious differences in gene expression between transgenic line and transgenic receptor during seed developmental stages. KEGG analysis found that the differentially expressed genes mainly annotated to metabolic pathways, such as carbohydrated metabolism and lipid metabolism. A 2-year single-location field trial revealed that three transgenic lines overexpressing *GmWRI1a* (GmWRI1a-OE) showed a stable increase in seed oil content of 4.97–10.35%. Importantly, no significant effect on protein content and yield was observed. Overexpression of *GmWRI1a* changed the fatty acid composition by increasing the linoleic acid (C18:2) content and decreasing the palmitic acid (C16:0) content in the seed. The three GmWRI1a-OE lines showed no significant changes in agronomic traits. The results demonstrated that the three *GmWRI1a* overexpression lines exhibited consistent increases in seed oil content compared with that of the wild type and did not significantly affect the seed yield and agronomic traits. The genetic engineering of *GmWRI1a* will be an effective strategy for the improvement of seed oil content and value in soybean.

## 1. Introduction

The demand for vegetable oils is increasing, owing to rising consumption for food uses and biodiesel production [1]. By 2050, the global demand for vegetable oils is expected to be more than double the current production [2]. Therefore, enhancing the oil content of oilseed crops without expansion of the cultivation area is an appropriate strategy to enhance oil yield. Soybean (*Glycine max* (L.) Merr.) is the world’s second-largest source of vegetable oil and accounts for 27% of global vegetable oil production [3]. The highest reported seed oil content is 27.9% within the U.S. Department of Agriculture soybean germplasm collection, which is relatively low compared with that of sesame (60%), although the lipid biosynthetic pathway is similar [4,5]. Thus, there is scope for using genetic engineering to elevate the seed oil content in soybean.

Improvement of oil traits by conventional plant breeding methods, such as pure-line selection and mutation breeding, is limited by the genetic variation currently available in soybean germplasm. Furthermore, oil yield is a quantitative trait controlled by multiple genes and is strongly influenced by the environment, and hence is difficult to manipulate [6,7]. The identification of genes associated with lipid biosynthesis and its regulation has enabled the application of genetic engineering strategies to enhance the seed oil content of soybean. Overexpression of a single gene that encodes an enzyme of the fatty acid and triacylglycerol (TAG) biosynthetic pathways may not achieve significant enhancement in seed oil yield [8,9,10]. In contrast, transcription factors, which simultaneously regulate the activities of numerous enzymes in the fatty acid biosynthesis pathway, have triggered considerable research interest [11,12,13,14,15].

WRINKLED 1 (WRI1) is an APETALA2/ethylene-responsive-element-binding protein (AP2/EREBP) subfamily transcription factor that directly or indirectly targets several enzymes involved in the late glycolysis and plastidial fatty acid biosynthetic network [16,17,18]. In *wri1* mutant lines of Arabidopsis, the lack of transcriptional activation of the fatty acid biosynthetic pathway in early maturing embryos results in an 80% reduction in seed oil content and wrinkled seeds [19]. However, overexpression of *AtWRI1* or other *WRI1* orthologs leads to an increase in seed oil content in transgenic plants, such as Arabidopsis, *Brassica napus*, *Camelina sativa*, *Zea mays*, *Brachypodium distachyon*, and *Glycine max* [17,20,21,22,23,24,25,26,27]. This effect suggests that the role of *WRI1* in oil accumulation is highly conserved in monocotyledonous and dicotyledonous plants. Thus, these studies offer a strategy to enhance oil synthesis by means of *WRI1* engineering.

To date, the effectiveness of increasing the seed oil content of soybean by overexpression of *GmWRI1* has been variable [26,27,28]. However, these studies only reported data from 1 year for seed oil content in transgenic soybean. To produce a new, genetically stable germplasm with high oil content to incorporate into soybean breeding programs and to demonstrate consistent elevation in oil content, stable transgenic soybean plants overexpressing *GmWRI1a* (GmWRI1a-OE) were generated in this study. The molecular genetic stability of the plants and the stability of the enhanced oil traits were analyzed, the transcriptomes of the transgenic line was sequenced and compared with wild-type seeds, and the phenotype of GmWRI1a-OE soybean lines derived from three transgenic events were observed in a single-location field trial over 2 years. Three stable transgenic lines were selected from five transgenic events, and homozygous T_4_ populations were raised. Bioassays confirmed that each of these stable transgenic lines continued to display significant elevation in seed oil content. The data presented support the viability of breeding for increased seed oil content by enhancing *GmWRI1a* expression in soybean.

## 2. Results

### 2.1. Identification of Transgene-Positive Plants and Quantification of Inserted Copy Number

**Figure 1:**
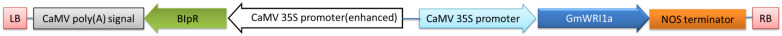
Schematic map of plant expression gene construct pCAMBIA3300-121-GmWRI1a. LB/RB, left/right T-DNA border sequences; BIpR, coding region of the phosphinothricin acetyl-transferase gene driven by enhanced CaMV *35S* promoter; GmWRI1a, coding region of the *GmWRI1a* gene driven by CaMV *35S* promoter.

**Figure 2:**
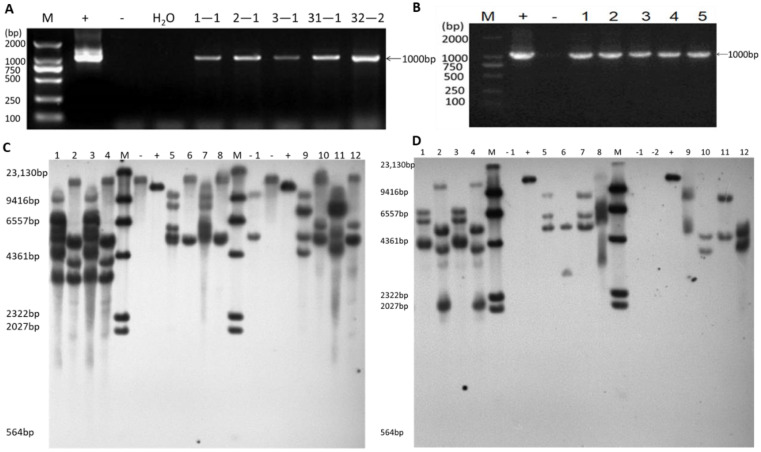
Molecular biology identification of GmWRI1a-OE lines. (**A**) PCR amplification of *bar* and CaMV *35S* promoter (1000 bp) from the genomic DNA of T_1_ transgenic plants. Lane M is 2000 bp molecular weight marker ladder; Lane—is WT plant (negative control); Lane+ is pCAMBIA3300-121-GmWRI1a (positive control); Lane H_2_O is water (negative control). (**B**) PCR amplification of *bar* and CaMV *35S* promoter (1000 bp) from the genomic DNA of T_2_ transgenic plants; Lanes 1–5 are 1-1, 2-1, 3-1, 31-1, and 32-2, respectively; Lane M is 2000 bp molecular weight marker ladder; Lane—is WT plant (negative control); Lane+ is pCAMBIA3300-121-GmWRI1a (positive control). (**C**) Southern blotting analysis of the T_3_ and T_4_ transgenic lines after digesting genomic DNA with *Hind* III and *EcoR* I, and probing with digoxigenin labeled *GmWRI1a*. Lanes 1, 5, and 9 are genome DNA of T_3_ generations 3-1, 31-1, and 32-2 digested by *Hind* III, respectively; Lanes 2, 6, and 10 are genome DNA of T_3_ generations 3-1, 31-1, and 32-2 digested by *EcoR* I, respectively; Lanes 3, 7, and 11 are genome DNA of T_4_ generations 3-1, 31-1, and 32-2 digested by *Hin*d III, respectively; Lanes 4, 8, and 12 are genome DNA of T_4_ generations 3-1, 31-1, and 32-2 digested by *Eco*R I, respectively; Lane M is 23 kb DNA marker; Lane -1 is genome DNA of DN50 digested by *Hind* III; Lane—is genome DNA of DN50 digested by *Eco*R I; Lane+ is positive plasmid. (**D**) Southern blotting hybridization analysis of the T_3_ and T_4_ generation transgenic soybean lines after digesting genomic DNA with *Hin*d III and *Eco*R I and probing with digoxigenin-labeled *bar*. Lanes 1, 5, and 9 are genome DNA of T_3_ generations 3-1, 31-1, and 32-2 digested by *Hin*d III,, respectively; Lanes 2, 6, and 10 are genome DNA of T_3_ generations 3-1, 31-1, and 32-2 digested by *Eco*R I, respectively; Lanes 3, 7, and 11 are genome DNA of T_4_ generations 3-1, 31-1, and 32-2 digested by *Hin*d III, respectively; Lanes 4, 8, and 12 are genome DNA of T_4_ generations 3-1, 31-1, and 32-2 digested by *Eco*R I, respectively; Lane M is a 23 kb DNA marker; Lane-1 is genome DNA of DN50 digested by *Hin*d III; Lane-2 is genome DNA of DN 50 digested by *Eco*R I; Lane+ is positive plasmid.

**Figure A1:**
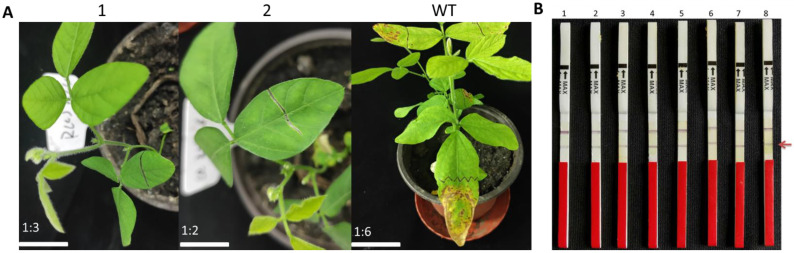
The identification of Basta resistance in transgenic plants. (**A**) Basta painting of T_0_ generation transgenic plant in the greenhouse using a 1:1000 diluted solution of the herbicide Basta (135 g L^−1^). (**B**) LibertyLink strip analysis of T_0_ generation transgenic plants; *lanes* 1–8 are transgenic plants.

Cotyledons from the conventional soybean cultivar Dongnong50 (DN50) were transformed via an *Agrobacterium*-mediated soybean cotyledonary node transformation system, with the *35S:GmWRI1a* construct (Figure 1). Eight transgene-positive T_0_ plants were identified by painting fully expanded leaves with a 1:1000 diluted solution of the herbicide Basta (135 g L^−1^) (Figure A1A) and the LibertyLink strip (Figure A1B). The specific PCR products of the *bar* gene and CaMV *35S* promoter region (about 1000 bp) were amplified from the T_1_ and T_2_ transgenic plants (Table A1). However, under the same conditions, no PCR products were amplified for DN50. These results indicated that *bar* was transformed into soybean and was stably inherited by the progeny (Figure 2A,B). Three GmWRI1a-OE lines (3-1, 32-2, and 31-1) were selected to evaluate the effect of *GmWRI1a* insertion in the soybean genome. Stable integration of *GmWRI1a* and *bar* in the genome was further confirmed by Southern blotting analysis of T_3_ and T_4_ GmWRI1a-OE lines. Probing with *GmWRI1a*, there was three copies of 3-1 line after subtracting the copy number produced by DN50 when the genomic DNA was digested with *Hind* III and *EcoR* I, respectively; two copies and one copy of 31-1 line, respectively; and two copies of 32-2 line (Figure 2C). Furthermore, probing with *bar*, there was three copies and four copies of 3-1 line when the genomic DNA was digested with *Hind* III and *EcoR* I, respectively; three copies and two copies of 31-1 line, respectively; and two copies of 32-2 line (Figure 2D). The results showed that *GmWRI1a* and *bar* were integrated into the genome with low copy numbers and were stably transmitted to the next generation. 

### 2.2. Identification of Integration Sites in GmWRI1a-OE Lines 3-1 and 31-1

To identify the putative insertion sites and the flanking sequences of the transgene as well as of partial vector fragments, whole genome sequencing and genetic analysis of the 3-1 and 31-1 transgenic lines were performed. The BLAST search results showed that the T-DNA fragment was likely inserted at 35,103,524 bp to 35,103,548 bp on chromosome 05 in transgenic line 3-1, which was a genomic spacer region between Glyma.05G158800.1 and Glyma.05G1589000.1. Moreover, the T-DNA fragment was likely inserted at 2,505,198 bp to 2,505,205 bp on chromosome 12 in transgenic line 31-1, which was near the upstream region of the promoter of Glyma.12G033200. Both insertion sites did not affect coding regions of functional genes.

**Figure 3:**
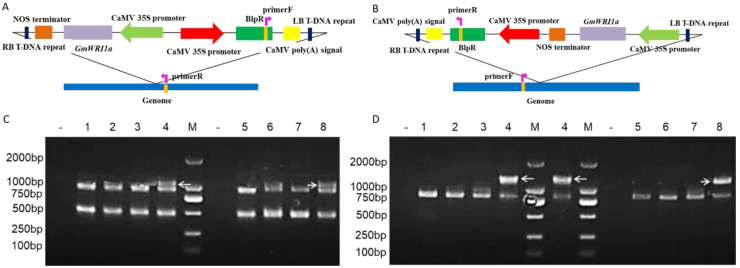
Integration sites of the T-DNA in 3-1 and 31-1 soybean. (**A**) Location of primers designed for event-specific PCR detection of 3-1. (**B**) Location of primers designed for event-specific PCR detection of 31-1. (**C**) Confirmation of the integration sites of the 3-1, predicted on the basis of DNA sequencing and data analysis results; Lane—is water (negative control); Lane M is 2000 bp molecular weight marker ladder; Lanes 1–4 are PCR amplification of *bar* and flanking region from the genomic DNA of T_3_ generations 1-1, 31-1, 32-2, and 3-1 respectively; Lanes 5-8 are PCR amplification of *bar* and flanking region from the genomic DNA of T_4_ generations 1-1, 31-1, 32-2, and 3-1, respectively. (**D**) Confirmation of the integration sites of 31-1, predicted on the basis of DNA sequencing and data analysis results; Lane—is water (negative control); Lane M is 2000 bp molecular weight marker ladder; Lanes 1–4 are PCR amplification of *bar* and flanking region from the genomic DNA of T_3_ generations 1-1, 3-1, 32-2, and 31-1, respectively; Lanes 5–8 are PCR amplification of *bar* and flanking region from the genomic DNA of T_4_ generations 1-1, 3-1, 32-2, and 31-1, respectively.

**Figure A2:**
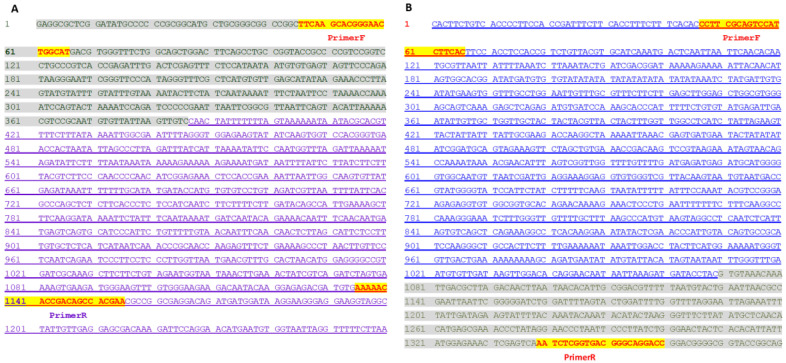
Sequencing results of PCR amplification product of *bar* and flanking region from genome DNA of 3-1 (**A**) and 31-1 (**B**) soybean. *Bar* gene’s sequences are in gray, genome DNA sequences are underlined, primers are highlighted in yellow.

To confirm the integration sites, PCR primers to amplify short fragments containing the genomic sequences flanking and the *bar* gene from 3-1 and 31-1 genomic DNA were designed, and then PCR analyses were conducted (Figure 3A,B, Table A1). The specific PCR products were amplified from 3-1 and 31-1 genomic DNA, as expected. However, no specific product was amplified from the WT DNA template (Figure 3C,D). The sequences of the amplified fragments from 3-1 and 31-1 were identical to those expected from the vector sequences (Figure A2A,B). In combination with the Southern blotting results, we determined that four copies of the T-DNA fragment were integrated at one site in chromosome 05 in the transgenic line 3-1, and three copies of the T-DNA fragment were integrated at one site in chromosome 12 of the transgenic line 31-1. 

### 2.3. Expression Level of GmWRI1a and Bar in Transgenic Soybean Plants

**Figure 4:**
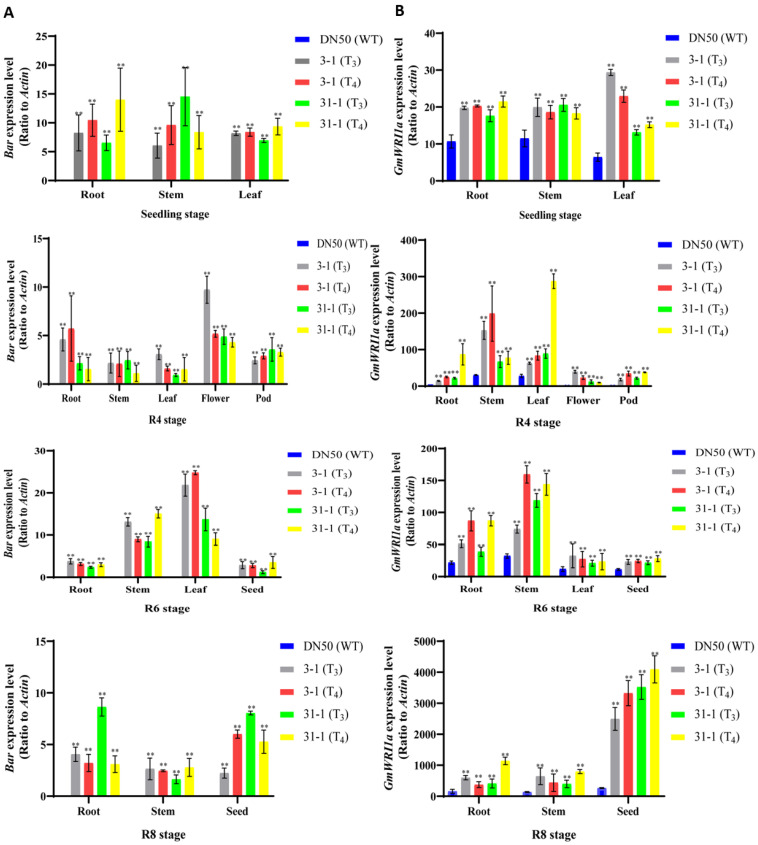
Relative expression level of *GmWRI1a* and *bar* genes in the different organs during development stages. Transgene expression was normalized using *GmActin4* gene and calibrated using the same sample under control conditions. Data shown represent the means ± SD of three independent experiments, with each experiment consisting of three technical replicates. Statistically significant differences between transgenic and WT plants are marked with asterisks (** *p* < 0.01; ANOVA). (**A**) The relative expression level of the *bar* gene in the different organs at four development stages from the T_3_ and T_4_ generation 3-1 and 31-1 soybean. (**B**) The relative expression level of the *GmWRI1a* gene in the different organs at four development stages from the T_3_ and T_4_ generation 3-1 and 31-1 soybean.

Different organs at different developmental stages from T_3_ and T_4_ GmWRI1a-OE plants (lines 3-1 and 31-1) and the WT were collected for *GmWRI1a* and *bar* expression analysis (Figure 4A,B). *GmWRI1a* and *bar* were expressed in the root, node, leaf, flower, and pod at all developmental stages of T_3_ and T_4_ transgenic plants. The expression level of *GmWRI1a* was lowest at the seedling stage and thereafter increased in the 3-1 and 31-1 lines. At the R8 developmental stage, the expression level of *GmWRI1a* peaked in the seed of the 3-1 and 31-1 lines (Figure 4B). The increased expression of *GmWRI1a* in the seed at stage 8 in the transgenic lines 3-1 and 31-1 accorded with the function of *WRI1*, which regulates fatty acid biosynthesis and shows the highest expression level at advanced stages of seed development. The *bar* expression level in all organs at all developmental stages in the 3-1 and 31-1 lines was lower than that of *GmWRI1a*. The *bar* expression level was lowest at stage R4 among all developmental stages in the 31-1 line (Figure 4A). In contrast, the *bar* expression level was lowest at stage R8 in the 3-1 line. The increased expression of *GmWRI1a* and *bar* in the 3-1 and 31-1 transgenic lines might have been caused by the CaMV *35S* promoter, which activated the genes constitutively. 

**Figure 5:**
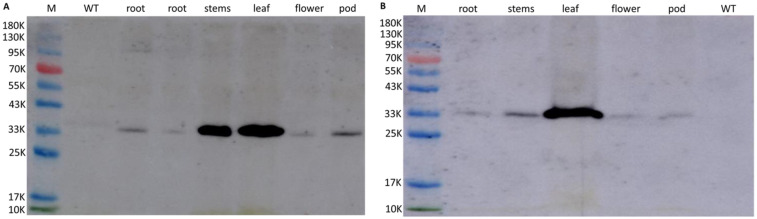
Western blotting analysis of the expression of BAR protein from the different tissues in T_4_ plants 3-1 (**A**) and 31-1 (**B**). Lane M is ladder marker; lane WT is leaf of wild-type soybean.

To further confirm that the target genes were transformed into the genome and were expressed stably, accumulation of the BAR polypeptide in different organs of the 3-1 and 31-1 transgenic lines was verified by visualization of the band in an immunoblot analysis with antibodies to BAR, whereas the immunoreactive band was not detected among proteins isolated from the leaves of the WT (Figure 5A,B). These results indicated that *GmWRI1a* and *bar* were transformed into soybean and were expressed stably.

### 2.4. Transcriptome Sequencing Analysis

**Figure 6:**
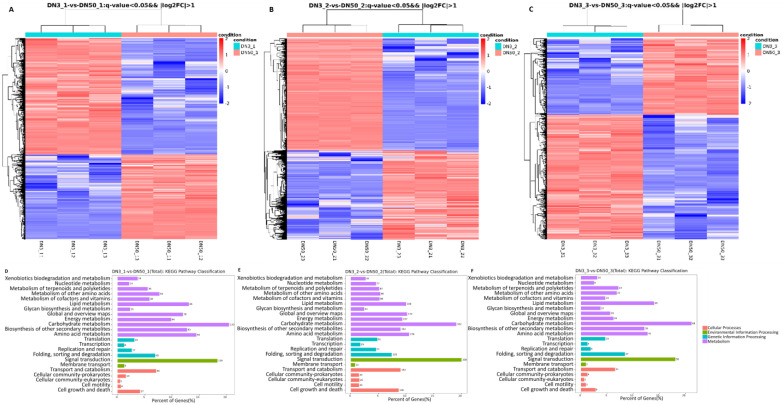
Differential gene expression transcriptome analysis. (**A**–**C**) Hierarchical cluster analysis of differential gene expression between 3-1 and DN50 at 21DAF, 28DAF, and 42DAF, respectively. Gene expression levels are shown using different colors, where blue represents a low expression level and red represents a high expression level. (**D**–**F**) KEGG pathway classification of differentially expressed genes between 3-1 and DN50 at 21DAF, 28DAF, and 42DAF, respectively.

To investigate whether gene expression within transgenic soybean seed tissue is altered, RNA-Seq was used to survey gene expressoin in 3-1 and DN50 at three seed developmental stages. After raw read quality filtering, 127.31 Gb of clean sequence data (6.78–7.42 Gb clean reads for each sample) were obtained for nine samples. Using the soybean DN50 transcriptome as a reference genome, 94.58–96.82% of the clean reads were mapped to the reference genome. There were 3901, 9401, and 1743 differentially expressed genes at the 21 DAF, 28 DAF, and 42 DAF seed developmental stages, respectively, for transgenic soybean and the transgenic receptor DN50. To illustrate differences between the lines, heat maps were constructed showing expression values for 3901, 9401, and 1743 of the most differentially expressed genes at 21 DAF, 28 DAF, and 42 DAF deveolpmental stages in the transgenic soybean versus nontransgenic comparion (Figure 6A–C). Expression levels of the top differentially expressed genes in the transgenic line were different from that of the wild type at three seed developmental stages.

KEGG (Kyoto Encyclopedia of Genes and Genomes) pathway analysis showed that the differentially expressed genes were mainly involved in the cellular processes, environmental information processing, genetic information processing, and metabolism. Most of the differentially expressed genes belonged to metabolism pathways at three seed developmental stages. At 21 DAF, there were 133 differentially expressed genes involved in carbohydrated metabolism and 85 genes involved in lipid metabolism (Figure 6D). At 28 DAF, there were 322 differentially expressed genes involved in carbohydrated metabolism and 169 genes involved in lipid metabolism (Figure 6E). At 42 DAF, there were 68 diffrentially expressed genes involved in carbohydrated metabolism and 43 genes involved in lipid metabolism (Figure 6F). Moreover, the differentially expressed genes were also involved in signal transduction, amino acid metabolism, and biosynthesis of other secondary metabolisms.

### 2.5. Seed Oil Content of Transgenic Plants Overexpressing GmWRI1a 

**Figure 7:**
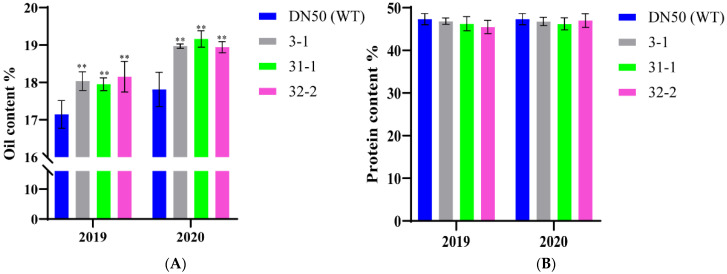
Seed oil contents (**A**) and seed protein contents (**B**) of three GmWRI1a-OE lines and wild-type DN50 over 2 seasons. The data represent the means ± SD of three replicate experiments, and the values are in dry weight (DW) for seeds, ** indicate significant differences compared with the wild type at *p* < 0.01, respectively, as determined by one-way analysis of variance (ANOVA).

The oil and protein contents of T_3_ and T_4_ homozygous GmWRI1a-OE plants grown under field conditions were measured and compared with those of WT plants at the Northeast Agricultural University Transgenic Biosafety Station in 2019 and 2020. Considering both generations, the seed oil content was increased by 4.97–10.35% in the three GmWRI1a-OE lines, which indicated generational stability of the seed oil phenotype (Figure 7A). An encouraging finding was that no major effect on protein content was observed in seeds of the transgenic lines (Figure 7B), which indicated that the transgene-associated increase in oil content did not cause a corresponding (1:1) decrease in protein content, in contrast to previous results obtained from traditional breeding.

To verify whether overexpression of *GmWRI1a* affects the fatty acid composition, the major fatty acid composition in seeds between the GmWRI1a-OE lines and WT plants were compared. A significant increase in linoleic acid (C18:2) content (*p* < 0.01) and a significant decrease in palmitic acid (C16:0) content (*p* < 0.01) were observed in seeds of the three GmWRI1a-OE lines (Table 1). These results suggested that overexpression of *GmWRI1a* increased the total oil content and changed the fatty acid composition in seeds of the three GmWRI1a-OE lines. 

### 2.6. Agronomic Parameters under Field Conditions

**Figure 8:**
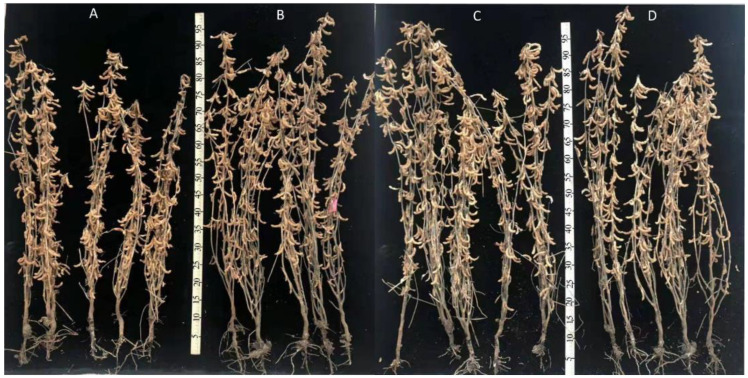
Representative pictures showing plant architecture of the homozygous T_4_ generation plants and WT at harvest stage. (**A**) WT plants; (**B**) 3-1 plants; (**C**) 31-1 plants; (**D**) 32-2 plants.

To determine whether *GmWRI1a* overexpression altered the growth and agronomic traits of the transgenic lines, the three GmWRI1a-OE lines and WT plants were grown at the Transgenic Experimental Field of Northeast Agricultural University for 2 years (Figure 8). The results showed that the plant height, pod number, and seed number per plant of 32-2 (T_4_) line and the seed weight per 100 seeds of 31-1 (T_4_) line were altered compared with those of the WT in this study (Table 2). However, these agronomic traits did not show continuous and stable changes according to two-generation data. Therefore, we could not determine that *GmWRI1a* overexpression altered the agronomic traits of 32-2 and 31-1 transgenic lines.

## 3. Discussion

For enhancement of plant lipid production, three types of genetic engineering strategies can be employed, categorized as a ‘Push’ strategy (upregulation of fatty acid), a ‘Pull’ strategy (increasing TAG assembly), and the ‘Accumulation’ approach (enhancing TAG storage or inhibiting TAG breakdown) [29]. Overexpression of *WRI1* is pivotal in the strategy, acting to upregulate (push) the de novo fatty acid pathway using a crucial transcription factor, combined with pulling the precursors toward the end products using rate-limiting enzymes, packaging TAG in oil bodies, and protecting TAG from degradation [30,31,32]. Transgenic plants overexpressing *AtWRI1* or *WRI1* orthologs have shown a significant increase in oil content in a number of studies [17,20,21,24,25,33,34]. In the present study, we developed three genetically stable transgenic soybean lines that overexpressed *GmWRI1a*. PCR and Southern blotting analysis of T_3_ and T_4_ transgenic lines confirmed that *GmWRI1a* had been integrated into the genome of transgenic receptor. The integration sites of the transgene cassette in the transgenic lines were rapidly identified by high-throughput sequencing of the genome. The foreign T-DNA integration sites of the 3-1 and 31-1 transgenic lines appeared to be genomic spacer regions, which did not affect coding regions of functional genes. This may have been the reason that GmWRI1a-OE plants were morphologically similar to WT plants under field conditions (Table 2). In combination with the results of Southern blotting, we determined that four copies of T-DNA fragments were integrated at one site in chromosome 05 in the 3-1 line, and three copies of T-DNA fragments were integrated into one site in chromosome 12 in the 31-1 line. Usually, genetic transformation using *A. tumefaciens* inserts a low number of copies in the host genome [35,36,37]. Low-copy-number transgenic events that show promising results for improvement of seed oil content are suitable for incorporation in breeding programs. Transformation events involving few transgene copies facilitate segregation in crosses to introduce a trait of interest into high-yielding cultivars aimed at the development of new commercial cultivars [38]. 

Using qRT-PCR, the expression levels of *GmWRI1a* and *bar* varied in different organs at different developmental stages in the transgenic lines, even though the genes were driven by the CaMV *35S* promoter in all lines. Significantly higher expression levels of *GmWRI1a* were observed in the transgenic lines compared with that of the WT at advanced stages of seed development, which was consistent with the important role of *GmWRI1* in soybean seed oil accumulation [28]. The expression levels of bar were higher at the seedling stage and lower at the R8 stage, which implied that *bar* is not involved in seed development. The reduced expression level of bar in the seed is favorable for safe human consumption of the seeds. 

Previously, Chen et al. introduced the *GmWRI1a* gene driven by the seed-specific napin promoter into soybean cultivar Williams 82, and transgenic soybean seeds grown in greenhouse showed a significant increase compared to the WT [26]. In this study, the three genetically stable transgenic soybean lines, using the stronger CaMV *35S* promoter to drive *GmWRI1a* expression, showed a significant increase in seed oil content. Moreover, the *GmWRI1a* was introduced into soybean cultivar DN50 that is a widely planted soybean cultivar in Northeast of China. The seed oil content in T_3_ plants was significantly higher than that of WT seeds. The seed oil content in the subsequent generation (T_4_) was similar to that in the preceding generation. Overexpression of *GmWRI1a* led to a more than 10% increase in seed oil content with no significant effect on protein content. So, the genetically stable transgenic lines obtained in this study could be directly applied to high-oil soybean breeding in Northeast of China. 

In addition, overexpression of *GmWRI1a* changed the fatty acid composition of the seed. The transgenic seeds accumulated a higher content of linoleic acid (C18:2) than that of WT seeds. Unlike *ZmWRI1* and *WRI1* genes from other plant species [21]. Overexpression of *GmWRI1* also upregulates genes encoding proteins involved in the late steps of TAG assembly in the endoplasmic reticulum, such as phosphatidylcholine diacylglycerol cholinephosphotransferase and lysophosphatidic acid acyltransferase, which likely activate acyl editing and production of more highly unsaturated-fatty-acid-containing TAGs [28]. Vogel et al. reported that the introduction of a seed-specific expression cassette carrying *AtWRI1* into soybean led to levels of palmitate of up to approximately 20%, but no change in total oil levels [39]. However, An and Suh introduced the *AtWRI1* gene driven by a seed-specific *SiW6* promoter into *Camelina sativa*, wherein the expression of *AtWRI1* caused an increase in total seed oil content by approximately 14% in transgenic lines compared to WT [24]. Moreover, the levels of oleic acid (C18:1) and eicosenoic acid (C20:1) were decreased, but there was an increase in the levels of linoleic acid (C18:2) and linolenic acid (C18:3) in transgenic lines compared to WT [24]. Heterologous expression of the same gene in variance plants may result in different results [24,40]. Even though *AtWRI1* and *GmWRI1* are higher homologously [26], they are both genes with different regulatory pathways in Arabidopsis and soybean [17,26,28]. Although both of them were introduced into soybean, the results obtained were not exactly similar. The exact differences and similarities of WRI1 transcriptional machinery among various plants still need to be further studied in the future.

Guo et al. reported that overexpression of *GmWRI1b* resulted in changes in the branch number and plant height [27]. However, in the present study, we did not observe stable changes in phenotypic traits in the 2-year experimental data. The difference may have been due to environmental influences or different insertion sites of the T-DNA. *GmWRI1a* can cause changes in phytohormone content and thus affect phenotypic traits, which will require further multi-year trials in the future to identify. Nevertheless, considering that the GmWRI1a-OE transgenic plants were morphologically similar to WT plants under field conditions (Table 2), it is highly likely that these transgenic lines could be used as important lines to breed new soybean cultivars with high seed oil content in the future.

## 4. Materials and Methods

### 4.1. Plant Materials

Soybean seeds of cultivars ‘Dongnong50’ (DN50) were provided by Key Laboratory of Soybean Biology in Chinese Ministry of Education, China.

### 4.2. Transformation and Identification of Transgene-Positive Plants

The expression cassette containing *GmWRI1a* was constructed using the pBI121 and pCAMBIA3300 vectors. These vectors are under the control of the constitutive promoter *Cauliflower mosaic virus* (CaMV) *35S* and the nopaline synthase (NOS) terminator. Two marker genes are also present in the cassette structure: the *bar* gene, which encodes for phosphinothricin acetyl transferase (PAT) and confers resistance to the herbicide ammonium glufosinate, is used as a selective agent, and the *NPTII* gene, which encodes neomycin phosphotransferase and confers resistance to the antibiotic kanamycin, is used to select colonies containing the inserted transgene. The recombinant plasmid pCAMBIA3300-121-GmWRI1a was introduced into *Agrobacterium tumefaciens* strain EHA 105 by the freeze–thaw method and used for *Agrobacterium*-mediated transformation of the soybean cotyledonary node following a method described previously [9,41].

To detect transgenic plants harboring *GmWRI1a*, we performed LibertyLink strip analysis, Basta painting, PCR, and Southern blot analysis. The T_0_ transgenic plants were screened with Basta (135 g·L^−1^, 1:1000 (*v*/*v*) dilution); firstly, the regenerated plants that remained green compared with the WT were tested using the LibertyLink strip to determine the PAT protein content following the manufacturer’s instructions (Envirologix Inc., Portland, ME, USA). For T_1_ and T_2_ transgenic plants and wild-type soybean, PCR was conducted to amplify the *bar* gene and the CaMV *35S* promoter using Bar+35S-specific primers (Table A1) to confirm T-DNA insertion. Genomic DNA was isolated using the Genomic DNA Purification Kit (Transgen Biotech, Beijing, China). The thermal cycling protocol comprised an initial denaturation at 94 °C for 3 min, followed by 30 cycles at 94 °C for 30 s, 64 °C for 30 s, and 72 °C for 1 min. After testing with Basta-resistance and PCR amplification, three GmWRI1a-OE lines (3-1, 31-1, and 32-2) were selected to be further identified by Southern blot analysis. Genomic DNA (30 µg) isolated from young leaves of homozygous T_3_ and T_4_ lines was digested with the restriction enzymes *Eco*RI and *Hin*dIII, respectively, at 37 °C overnight. Digested DNA was separated in 0.8% (*w*/*v*) agarose gel and blotted onto a Hybond N^+^ nylon membrane (Bio-Rad, Hercules, CA, USA) for hybridization. The sequence-specific fragment of the *bar* and *GmWRI1a* was DIG-labeled as the probe using the DIG-High Prime DNA Labeling and Detection Starter Kit I (Roche, Mannheim, Germany) in accordance with the manufacturer’s protocol.

### 4.3. Confirmation of Insertion Sites and Flanking Sequences

High-throughput DNA sequencing with 25× genome coverage was performed by BioMarker (Beijing, China). Contigs containing vector sequences and their putative flanking sequences were identified by BLAST searches against databases using binary vector sequences as query sequences. The selected contigs were then used as query sequences in BLAST searches against the soybean Williams 82 reference genome sequence (version Wm82.a2.v1) in SoyBase (https://soybase.org/ [accessed on 8 October 2020]) to identify the chromosomal position and flanking sequences of the vector fragments, including the inserted T-DNA and vector backbone fragments. The identified sites were PCR-amplified from genomic DNA isolated from transgenic plants, and the PCR products were sequenced for confirmation (Table A1).

### 4.4. Analysis of Gene Expression by Quantitative RT-PCR and Western Blotting

Different organs from transgenic and WT plants were collected for expression analysis. Total RNA was isolated from seedlings using TRIzol Reagent (Invitrogen, Carlsbad, CA, USA) in accordance with the manufacturer’s protocol. The isolated RNA was treated with RQ1 RNase-free DNase l (Promega, Madison, WI, USA) to remove any contaminating DNA. Real-time quantitative RT-PCR (qRT-PCR) was performed using the Chromo4 Real-Time PCR System (ABI PRISM 7900HT) and the SYBR Green PCR Master Mix Reagent (Takara, Otsu, Japan). Soybean *GmActin4* was used as an internal control. The qRT-PCR protocol comprised 95 °C for 3 min, then 40 cycles of 95 °C for 5 s, and 60 °C for 30 s. The qRT-PCR reactions were performed following the manufacturer’s protocol with three biological and three technical replicates for each sample. The relative expression of the target gene was calculated using the 2^−ΔΔ*C*t^ method for all samples. All primers used are listed in Table A1.

Expression of the BAR protein in T_4_ plants was assessed by Western blot analysis. Total proteins isolated from young leaves were fractionated by dodecylsulphate-polyarylamide gel electrophoresis (SDS-PAGE) [42] using a Mighty Small II electrophoresis system (Hoefer Scientific Instruments, San Francisco, CA, USA). The proteins were resolved on a slab gel (10 × 8 × 0.75 cm) comprising a 13.5% (*w*/*v*) separation gel and a 4% (*w*/*v*) stacking gel, and electroblotted onto a pure nitrocellulose membrane (Midwest-Scientific, Valley Park, MO, USA). Immunoblot analysis was performed using BAR protein antibodies produced by the Shanghai YouLong Biotechnology Co., Ltd. (Shanghai, China).

### 4.5. Transcriptome Sequencing and Data Analysis

Soybean samples were planted in a greenhouse. Developing seeds of 3-1 at 21 DAF, 28DAF, and 42DAF were collected for RNA sequencing (RNA-seq). Three seeds from each development stage were collected and processed individually, generating three biological replicates from each development stage and three biological replicates from each construct. Total RNA was extracted with the TIANGEN RNAprep Pure Plant Kit. A total of 15 ug of RNA per sample was used to sequence the transcriptome. RNA was fragmented and used as a template for first-strand cDNA synthesis using reverse transcriptase and random primers, followed by second-strand cDNA synthesis. End repair and adding an A base to the 3′ end were performed on the double-stranded cDNA, and DNA adapter was then ligated to the DNA fragment. Finally, the adapter-ligated DNA was enriched by 15 cycles of PCR and gel purified for Illumina single-end sequencing.

After adapter clipping and quality filter, the clean reads were aligned to the soybean DN50 reference transcriptome using HisHat (Kim et al., 2015). Gene expression levels were evaluated in reads per kb per million reads (RPKM) on the basis of the number of reads mapped to the reference sequence. Differential expression analysis was performed using DEGseq (Love et al., 2014). The screening conditions for differentially expressed genes were set at a q < 0.05 and foldchange > 2. Finally, the differentially expressed genes were mapped to GO terms and the KEGG database, and significantly enriched GO and KEGG terms were thus identified.

### 4.6. Field Trial Methods

The T_3_ and T_4_ GmWRI1a-OE lines (3-1 and 31-1) and soybean ‘Dongnong 50’ (DN50) plants were grown under natural conditions at the Transgenic Experimental Field of Northeast Agricultural University (45°45′06″ N, 126°43′21″ E), Harbin, China, in 2019 and 2020. The experiment employed a randomized complete block design (RCBD) with three replicates. Seeds were sown in rows 3 m long and 0.65 m apart, with 6 cm spacing between plants. After full maturity, mature seeds were harvested from the plants and air-dried.

In total, 10 WT plants and 10 individuals of each transgenic line per accession were randomly sampled in each replication at the mature stage; thus, 30 plants for each accession were used for phenotypic analyses. Plant height, number of primary branches, number of primary internodes, pod number, seed number per plant, seed weight per plant, and 100-seed weight were measured. The experiments were performed with three replications.

### 4.7. Analysis of Seed Oil and Protein Contents

The seed oil content was analyzed using the Soxhlet extraction method [9,14]. The seed protein content was analyzed using the Kjeldahl method [43]. The total oil and protein contents were expressed on a percentage dry-weight basis. 

### 4.8. Fatty Acid Composition

Powdered seeds (0.1 g) were used to analyze the fatty acid composition by gas chromatography (GC-14C, Shimadzu Company, Tokyo, Japan). Fatty acid extraction and analysis were performed in accordance with the method of Zhang et al. [44]. All test columns had nominal dimensions of 30 × 0.125 m with 0.13 μm film thickness. Operating conditions were as follows: carrier, hydric (400 mL min^−1^) split injection, injection temperature 250 °C, detector temperature 250 °C, and column temperature 210 °C [45,46]. 

### 4.9. Statistical Analysis

Data analyses were performed using IBM SPSS Statistics 19 (IBM, Armonk, NY, USA) and EXCEL 2010. A one-way analysis of variance (ANOVA) was used to compare the treatment means. The data are presented as means ± standard deviations (SD).

## 5. Conclusions

In this study, we generated three GmWRI1a overexpression lines, which exhibited consistent increases in seed oil content compared with that of the WT. The enhanced seed oil content remained stable and did not significantly affect the seed yield and agronomic traits. The three GmWRI1a overexpression lines increased unsaturated fatty acid (C18:2) content and decreased saturated fatty acid (C16:0) content in the seed. The transgenic soybean lines generated in this study showed potential for use in soybean oil production and breeding in the future.

## Figures and Tables

**Table 1:** Fatty acid composition of GmWRI1-OE lines and DN50.

Lines	C16:0	C18:0	C18:1	C18:2	C18:3
DN50	11.08 ± 0.02	3.46 ± 0.01	23.68 ± 0.09	49.71 ± 0.13	9.18 ± 0.03
3-1	10.95 ± 0.01 **	3.36 ± 0.02 **	20.13 ± 0.09 **	52.71 ± 0.08 **	9.85 ± 0.04 **
31-1	10.82 ± 0.02 **	3.49 ± 0.03	23.58 ± 0.12	50.06 ± 0.08 **	8.96 ± 0.06 **
32-2	10.40 ± 0.02 **	3.06 ± 0.01 **	22.90 ± 0.02 **	50.90 ± 0.06 **	9.70 ± 0.04 **

The data represent the means ± SD of the three replicate experiments, and the values were reported as the relative percentage of individual fatty acid in the total fatty acids. C16:0, C18:0, C18:1, C18:2, and C18:3 represent palmitic, stearic, oleic, linoleic, and linolenic acids, respectively. ** indicates significant differences compared with the wild type as determined by one-way ANOVA (*p* < 0.01).

**Table 2:** Agronomic performance of GmWRI1a-OE lines and WT plants in field.

Genotype	Plant Height (cm)	Number of Primary Internode	Number of Primary Branches	Pod Number Per Plant	Seed Number Per Plant	Seed Weight Per 100 Seeds (g)
DN50 (WT)	84.02 ± 12.84	18.59 ± 2.14	3.31 ± 2.24	117.84 ± 63.38	257.79 ± 26.97	7.56 ± 0.77
3-1 (T_3_)	88.67 ± 5.70	18.59 ± 2.17	2.58 ± 2.17	90.33 ± 15.60	259.81 ± 18.42	7.72 ± 1.26
3-1 (T_4_)	88.92 ± 8.53	18.79 ± 1.53	3.29 ± 2.16	104.50 ± 14.76	245.89 ± 11.12	8.45 ± 0.86
31-1 (T_3_)	86.00 ± 7.10	18.74 ± 1.71	2.59 ± 2.02	98.71 ± 47.10	206.25 ± 21.89	8.02 ± 1.23
31-1 (T_4_)	87.12 ± 5.04	19.00 ± 1.22	2.74 ± 1.29	91.12 ± 25.48	207.73 ± 18.69	9.70 ± 1.06 **
32-2 (T_3_)	85.33 ± 8.93	19.00 ± 1.05	3.40 ± 2.24	113.83 ± 43.54	256.06 ± 25.90	8.62 ± 3.64
32-2 (T_4_)	70.10 ± 8.38 **	18.87 ± 1.41	2.10 ± 1.69	74.73 ± 21.98 **	157.61 ± 10.87 **	8.43 ± 1.48

Values are means ± SD of three replicates. A total of 30 plants were measured in each independent measurement. ** is significantly different from the wild type as determined by one-way ANOVA (*p* < 0.01).

## Data Availability

All data generated and analyzed in this study are included in this paper.

## Appendix B

**Table A1:** Primer pairs used in the study.

GmWRI1a-P1	5′-ACTGCTGGATCCATGAAGAGGTCTCCAGCATC-3′
GmWRI1a-P2	5′-CGTGCGACTAGTTCATAGATCTAGAGCATAGTCAC-3′
35s+bar-F	5′-TGTGATAACATGGTGGAGCAC-3′
35s+bar-R	5′-AAATCTCGGTGACGGGCA-3′
GmWRI1a-probe-F	5′-GCCTAAGCATCCAAGGAGGA-3′
GmWRI1a-probe-R	5′-GATGATGCCTAGCAACCCCA-3′
BAR-target-F1	5′-GCGGTACCGGCAGGCTGAAG-3′
BAR-target-R1	5′-CCGCAGGAACCGCAGGAGTG-3′
Actin4-F	5′-GTGTCAGCCATACTGTCCCCATTT-3′
Actin4-R	5′-GTTTCAAGCTCTTGCTCGTAATCA-3′
QRTpcr-WRI1-F	5′-CACCACAGCAGCACCAAGTTC-3′
QRTpcr-WRI1-R	5′-TGCCCACCAAGTCATCATC-3′
QRTpcr-Bar-F	5′-GTCCAGTCGTAGGCGTTGC-3′
QRTpcr-Bar-R	5′-GTCTGCACCATCGTCAACCA-3′
